# Enhancement of Thermal and Mechanical Properties of Bismaleimide Using a Graphene Oxide Modified by Epoxy Silane

**DOI:** 10.3390/ma13173836

**Published:** 2020-08-31

**Authors:** Hao Jiang, Yanyan Ji, Jiantuo Gan, Lei Wang

**Affiliations:** 1 School of Materials Science and Engineering, Xi’an Shiyou University, Xi’an 710065, China; jtgan@xsyu.edu.cn (J.G.); leiw@xsyu.edu.cn (L.W.); 2 Xi Jing Electric Corporation, Xi’an 710065, China; jiyanyan@xachuangyan.com

**Keywords:** graphene oxide, bismaleimide, thermomechanical, mechanical properties

## Abstract

A thermosetting resin system, based on bismaleimide (BMI), has been developed via copolymerization of 4,4′-diaminodiphenylsulfone with a newly synthesized graphene oxide modified using epoxy silane (ES-GO). The effect of ES-GO on the thermomechanical and mechanical properties of cured modified resin was studied. To evaluate the efficiency of the modified BMI systems, the composite samples using glass fiber cloth were molded and tested. Thermogravimetric analysis indicates that the cured sample systems displays a high char yield at lower concentrations of ES-GO (≤0.5 wt.%), suggesting an improved thermal stability. Using dynamic mechanical analysis, a marked increase in glass transition temperature (*Tg*) with increasing ES-GO content was observed. Analysis of mechanical properties reveals a possible effect of ES-GO as a toughener. The results also showed that the addition of 0.3 wt.% ES-GO maximizes the toughness of the modified resin systems, which was further confirmed by the result of analysis of fracture surfaces. At the same time, a molded composite with ES-GO showed improved mechanical properties and retention rate at 150 °C as compared to that made with neat resin.

## 1. Introduction

Bismaleimide (BMI) resins are widely applied in high-tech fields due to their high thermal stability, great mechanical properties and good moisture, radiation and corrosion resistance after curing [1,2,3,4,5,6]. However, wider usage of BMI resins has been limited because of the inherent brittleness caused by the high crosslink density and the aromatic nature of the network. Efforts have been devoted to overcome those problems, such as incorporating microphase of dispersed elastomers or thermoplastic polymers [7,8,9]; cocuring with other thermosetting materials [10,11,12]; decreasing the crosslink density through Michael addition with active hydrogen-containing compounds [13,14,15]; designing and synthesis of a new type of chain-extended BMI [16,17,18]. As far as the toughness is concerned, the above methods are effective but usually result in accompanying shortcomings, such as reduction in modulus or poorer thermomechanical properties [19,20,21]. Therefore, enhancing the toughness of BMIs without compromising but rather improving the related physical and chemical properties is a challenge; this area also needs to be further explored. At present, a strategy to solve the above problems is copolymerizing with nanomaterials, which may be the most preferable method for BMI modification.

To our knowledge, due to its hexagonal honeycomb lattice structure composed of sp² hybrid orbitals, graphene has peculiar and unique properties, such as superior tensile strength, high flexibility, mechanical property, good thermal conductivity, remarkable electron-transporting properties and so on [22,23,24,25,26,27,28,29,30]. Graphene oxide (GO) is an oxidized form of graphene, it has a similar structure as graphene, but its physical and chemical properties are quite different [31,32]. Due to the large number of oxygen functional groups, GO can be used as a new class of modifiers for resin matrixes, resulting in the observation of new properties or in a considerable improvement of them, leading to modification of structure-properties relations, which otherwise wouldn’t be detected [33,34,35]. So far as we know, the location of GO will become a stress concentration point during the deformation of the resin matrix, which causes the resin matrix around it to be yield, and then absorbing a large amount of deformation work, so as to achieve the purpose of toughening [36,37]; in addition, a large number of active functional groups can be introduced into the surface of GO via modifying, which can be both physically or chemically cross-linked with the polymer chain. When the material receives impact, the cross-linked network structure will tend to help the resin matrix to bear the external force and conduct the force to the GO through the molecular chain, so as to achieve the purpose of toughening the resin matrix [38,39]. Recently, there has been more focus on incorporation of GO into BMI networks [40,41,42,43,44,45]. However, scarce literature focused on improving toughness while improving thermomechanical properties has been reported.

Our current research deals with the design and synthesis of a functional GO modified by epoxy silane (ES-GO) which can be used for enhancing the performance of BMI. ES-GO was expected to be effective in the toughening and enhancement of heat resistance due to its sufficiently reactive sites of epoxy groups and thermal conductive graphene structure. The effects of ES-GO content on thermal stability and the mechanical properties of the modified BMI system were studied, and finally a complementary study of glass-fiber-reinforced composites based on modified resins was performed and is described in some detail.

## 2. Experimental

### 2.1. Raw Materials

4,4′-Bismaleimide biphenyl methane was purchased from the Fengguang Chemical Co., Ltd. (Wuhan, Hubei, China) and was used as received. 4,4-Diaminodiphenyl sulfone (DDS) was used as received from Aldrich Chemical (Milwaukee, WI, USA). Diallyl bisphenol A (DABPA) was purchased from the Fuchen Chemical Co., Ltd. (Tianjin, China). Graphite powder is an industrial product purchased from Jixihao New Energy Materials (Tianjin, China). 3-glycidyl ether oxypropyl trimethoxysilane was purchased from Shanghai Aladdin Biotechnology (Shanghai, China). Sodium nitrate, hydrogen peroxide solution, concentrated sulfuric acid, Concentrated hydrochloric acid and potassium permanganate were commercially supplied (Aladdin Biotechnology, Shanghai, China) and were used as received.

### 2.2. Preparation of Specimens

**Figure 1:**
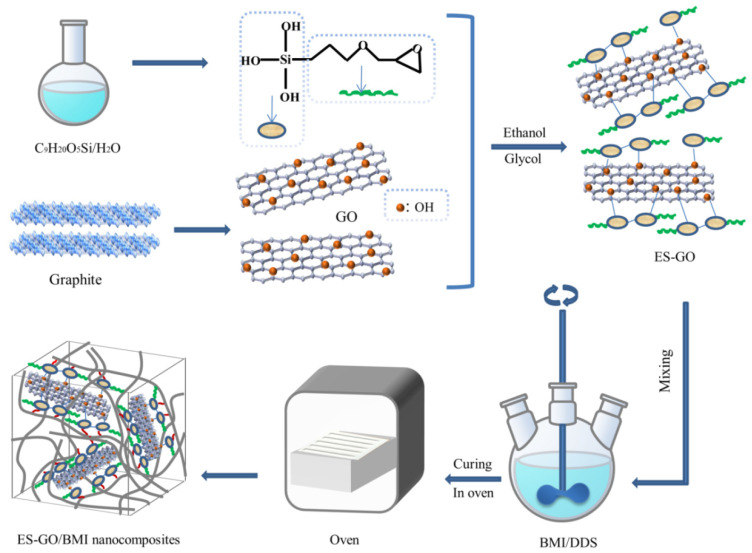
Schematic diagram for the preparation of epoxy silane modified graphene oxide (ES-GO)/Bismaleimide (BMI) nanocomposites.

100 mg graphene oxide was mixed into the solvent (80 mL ethanol + 20 mL glycol), and after ultrasonic treatment for 15 min, 25 mg 3-glycidyl ether oxypropyl trimethoxysilane was added into the graphene oxide suspension; then, the reaction was carried in water under a constant temperature of 85 °C for 5 h; when the reaction was over, the sediment was taken out of the reaction solution, which has left to stand, and separated. After dialyzing the resulting solution until a pH of about 7, the residual solid was obtained through a freeze-drying process. Finally, the epoxy silane modified graphene oxide (ES-GO) was obtained. The synthesis procedure is schematically illustrated in Figure 1.

BMI and DDS (molar ratio = 2:1) were mixed and melted while maintaining a low stream of nitrogen and agitation for 0.5 h. Then, DABPA was added into the mixture and mixed at 130 °C for 0.5 h; after that, ES-GO was placed into above mixture until it evenly dissolved in the system. Then, the mixture was poured into a mold which had been preheated and degassed in a vacuum oven at 135 °C. The curing process was performed as follows: 150 °C/2 h + 180 °C/2 h + 210 °C/2 h + 240 °C/4 h. The glass fiber cloth-reinforced composites were laminated by wet layup and cured using a hot press at a pressure of 2 MPa using a cycle of 150 °C/1 h + 165 °C/2 h + 180 °C/2 h.

### 2.3. Characterization and Measurements

Fourier transform infrared (FTIR) spectra of the samples were obtained using a WQF-310 FTIR spectrometer supplied by Optical Instrument Factory (Beijing, China). X-ray photoelectron spectroscopy (XPS, Thermal Scientific K-Alpha XPS spectrometer) was used to investigate the surface elemental composition. Thermogravimetric analysis (TGA) of the samples was performed on a Perkin-Elmer Pyris1 thermo gravimeter in inert atmosphere from 25 °C to 900 °C at a heating rate of 20 °C/min. Dynamic mechanical analyzers (TA DMA 2980) of the samples were performed in three-point bending modes using a TA DMA 2980 instrument. The support span was 50 mm and the heating rate of 2 °C/min at an oscillation frequency of 1 Hz was selected. Scanning electron microscope (SEM, SM-6700F) was employed to examine the morphologies of the fracture surfaces of the cured resin. The water absorption rates of cured resins were determined according to the standards GB/T 1462-2005. The mechanical properties of cured resins were measured according to GB/T 2567-2008. The mechanical properties of the composites were measured according to GB/T3356-2005 and JC/T 773-1996, respectively.

## 3. Results and Discussion

### 3.1. Characterization of ES-GO

**Figure 2:**
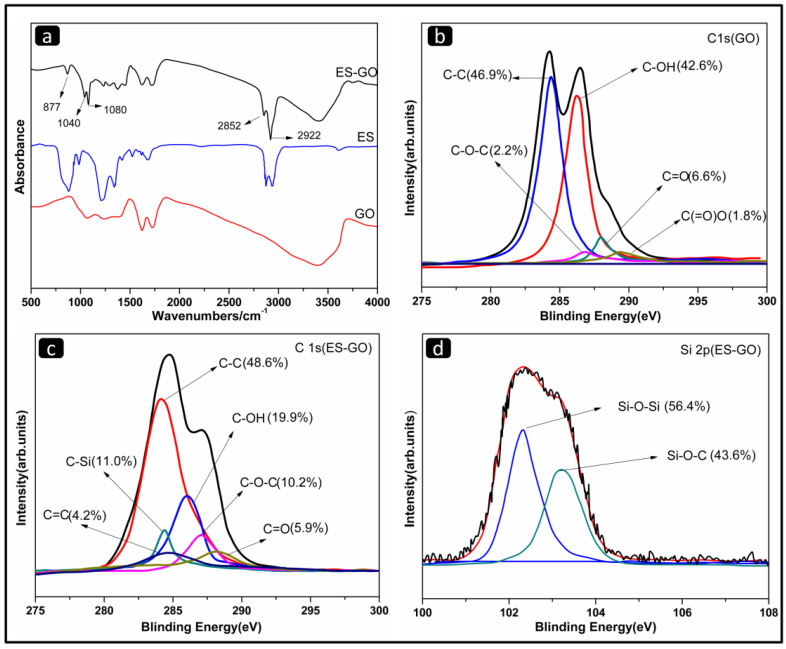
(**a**) Fourier transform infrared (FTIR) spectra of GO, ES and ES-GO;(**b**) C1s X-ray photoelectron spectroscopy (XPS) spectra of GO; (**c**) C1s XPS spectra of ES-GO; (**d**) Si2p XPS spectra of ES-GO.

The ES-GO was synthesized to modify the BMI/DDS resin system, and its structural information was given by FTIR spectra (Figure 2a).

The FTIR spectrum presents a comparison of GO, ES and ES-GO. In the case of ES-GO, the band at 3410 cm^−1^ became weaker than GO, which is attributed to the large consumption of O–H bonds; the characteristic peaks appearing at 2852 cm^−1^ and 2922 cm^−1^ are typically related to methyl and methylene groups, which originated from the alkyl chains assigning to silane moieties of ES-GO. In view, the obvious absorption peaks at 877 cm^−1^ correspond to Si–C bonds. The peak observed at 1040 cm^−1^ is related to the vibration bands of silicon oxygen groups (Si–O–Si), which can be ascribed to epoxy silane hydrolyzed and self-polymerized. In addition, a peak at 1080 cm^−1^ belongs to the Si–O–C stretching, which was weaker than the peak of the Si–O–Si stretching, evidencing that the self-polymerization of epoxy silane and the grafting on the surface of graphene oxide occur simultaneously. The appearance of Si–O–Si and Si–O–Si bonds indicate the successful chemical functionalization. The results confirmed the successful fabrication [46].

Figure 2b–d display the XPS information of GO and ES-GO nanocomposites respectively. They are applied to further analyze the bond state of the surface and the elementary composition. As can be seen from Figure 2b, the C1s peaks of GO are deconvoluted into five forms of carbon, including C–C (284.5 eV), C–OH (286.2 eV), C–O (287.5 eV), C=O (288.7 eV) and C(=O)O (289.4 eV). The XPS spectrum of ES-GO (Figure 2c) exhibits a new peak located in 284.4 eV, which is related to C–Si. In addition, the C–OH content of ES-GO showed a significant decrease compared with GO, which can be attributed to epoxy silane reaction with the hydroxyl groups on the surface of graphene oxide after hydrolysis (as shown in Figure 1) resulting in a decrease in C–OH content. As can be judged from Figure 2d, the Si2p peaks of ES-GO in XPS spectrum are deconvoluted into two forms, including Si–O–Si (102.3 eV) and Si–O–Si (103.5 eV), which indicates that ES molecules were grafted onto the GO surface. This is consistent with the results of FTIR analysis.

### 3.2. Thermal Stability Analysis

**Figure 3:**
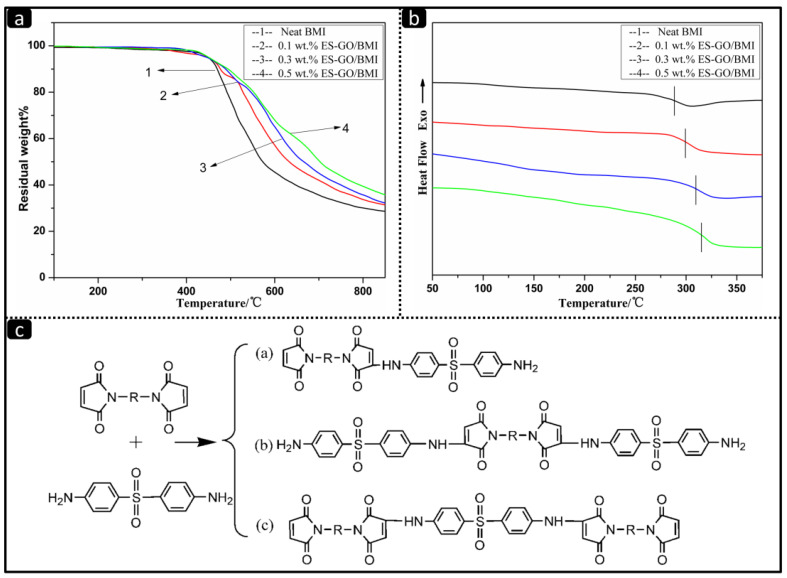
(**a**) Thermogravimetric analysis (TGA) curves of cured resin systems with different amount of ES-GO; (**b**) Differential scanning calorimeter (DSC) curves of cured resin systems with various amounts of ES-GO; (**c**) Proposed reactions in the process of 4,4-Diaminodiphenyl sulfone (DDS) chain extension of BMI.

The TGA curves of resin systems cured with various amounts of ES-GO are shown in Figure 3a; the TGA data are given in Table 1.

It can be seen that ES-GO has almost no effect on the initial decomposition temperature of the modified system. The mass loss in all cases starts at above 450 °C, which can be ascribed to the decomposition of the BMI network. However, the char yield of the modified BMI resin system at 850 °C slightly increases when the proportion of ES-GO is increased. The char yield shifts from 28.6% for neat resin to 35.6% for BMI modified with 0.5 wt.% of ES-GO. First of all, it can be attributed to the heat resistance of graphene oxide, which is much higher than the matrix resin. In addition, the ES-GO/BMI systems possess good thermal stability owing to the formation of a stable cross-linked structure which contains certain amino, carboxyl and other functional groups. To our knowledge, the thermal stability of BMI resin has been improved by ES-GO due to the reactions in the process of DDS chain extension of BMI, as shown in Figure 3c. All products of reaction and DDS can react with epoxy groups of epoxy silane, which ensure that ES-GO is not only an isolated lamellar substance present in the resin but also become part of the entire cross-linked macromolecular structure [47]. It can even be considered that ES-GO acts as a chain extender. In addition, part of ES-GO enters into the cross-linked network in the form of physical cross-links. The movement of graphene sheets is more difficult compared with the molecular chains; the existence of ES-GO limits the movement of molecular chains resulting in an improvement of thermal stability. The limitation is particularly obvious when the molecular chain motion is not strong under low temperature.

Figure 3b shows the differential scanning calorimeter (DSC) curves of cured BMI resin systems with the various additions of ES-GO. All cases show a single glass transition temperature (Tg) value which increases slightly with increasing ES-GO content. A similar trend for the heat distortion temperature (HDT) is also observed, as shown in Table 1. It can be seen from Table 1 that ES-GO has a positive effect on the HDT of cured modified resin, which is consistent with the aforementioned theory that ES-GO hinders the movement of molecular chains. When the movement of molecular chain is blocked, higher temperature and energy are needed to ensure the movement of molecular chain. Therefore, the HDT increases after ES-GO was added. When the load is so low, the effect it can have is negligible. At this time, the ES-GO content is too small to hinder the movement of the resin molecular chain; therefore, the HDT increases relatively in a slow pace with the increase of ES-GO loading at this stage. When the ES-GO loading is varied from 0.1 wt.% to 0.3 wt.%, the movement of molecular chains is gradually restricted with an increase in ES-GO content, which results in a significant increase in HDT. However, when the addition of ES-GO is more than 0.3 wt.%, excessive ES-GO began to agglomerate in the modified resin system, which reflects in a slow increase in the material’s HDT.

### 3.3. Dynamicmechanical Analysis

DMA is a widely used method in polymer characterization [48,49]. It measures the relation between the mechanical properties of viscoelastic materials and time, temperature or frequency. The sample is deformed by the action and control of periodic (sinusoidal) mechanical stress. The glass transition temperature (*Tg*) of the thermosetting resin represents the maximum service temperature, and it is an important physical parameter and also an important index for evaluating the heat resistance.

**Figure 4:**
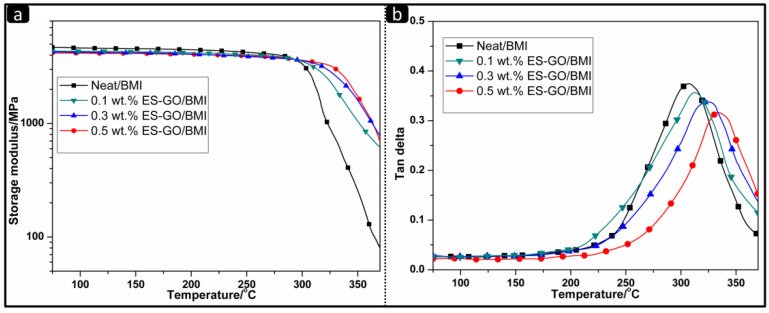
Storage modulus (**a**) and tan*δ* (**b**) of cured resin systems.

It is usually simpler and more accurate to use the DMA method for the measurement of *Tg*. *Tg* can be directly expressed by the mechanical loss factor tan*δ*. The *Tg* of the modified systems with different contents of ES-GO is shown in Table 1. Figure 4 shows the temperature dependence of storage modulus and loss tangent (tan*δ*) of modified resin systems.

It can be seen from Figure 4a that the storage modulus (E′) changes with the change of mechanical properties. When the transition is about to end, the storage modulus decreases to almost zero. No significant differences were observed in the storage modulus change curves of all systems at temperatures below 300 °C. The storage modulus of the modified system containing ES-GO is slightly lower than that of the unmodified system; when the temperature continues to rise, the maximum decrease rate of storage modulus for different content systems is inversely related to the content of ES-GO. It can be proved that the ES-GO containing resin system has a higher cross-linking density than the neat resin system by DMA analysis. The increase in crosslink density can further reduce the mobility of molecular chains and make the network structure stable. Due to ES-GO participating in the curing reaction and forming a cross-linked network structure with the BMI resin matrix, a difference between the elastic modulus and tensile strength of modified resin systems with different ES-GO contents was observed.

As shown in Figure 4b, the speed of *Tg* values first increase slowly when the amount of ES-GO is varied from 0 wt.% to 0.1 wt.%, reach a maximum when the amount of ES-GO is varied from 0.1 wt.% to 0.3 wt.% and afterwards decrease when the amount of ES-GO is varied from 0.3 wt.% to 0.5 wt.%. When the addition of ES-GO is less than 0.1 wt.%, the limited content of ES-GO is difficult to form architecture by reaction with BMI. The increase of *Tg* is only credited to the heat-resistant structure of ES-GO. Similar behavior has been reported in the literature [50]. When the amount of ES-GO is varied from 0.1 wt.% to 0.3 wt.%, ES-GO can be introduced into the network structure of resin via a covalent bond to form a body structure, while providing a heat-resistant structure in the resin system, hence reflecting in a fast increase in *Tg*, which is benefited from the crosslink density of the cured network increases. When the addition of ES-GO is more than 0.3 wt.%, excess ES-GO would not be able to connect to the cross-linked network via a covalent bond, since ES-GO has tendency to be saturated in the resin system. However, excessive ES-GO still produces a stacking effect in the heat-resistant structure, which can also improve the thermal stability of the modified system slowly. These results are consistent with TGA, DSC and HDT measurement.

### 3.4. Mechanical Properties

**Figure 5:**
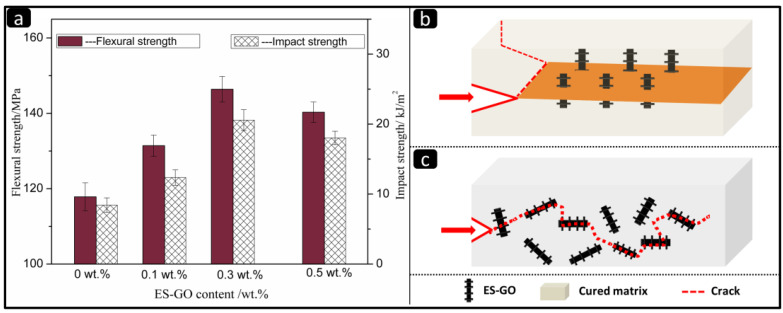
(**a**) Mechanical properties of the cured resins with different content of ES-GO; (**b**) mechanism of crack pining; (**c**) mechanism of crack deflection.

In order to study the effect of ES-GO on the mechanical properties of the cured resin, the impact of strength and flexural strength of the ES-GO/BMI composite were tested. As shown in Figure 5, it can be seen that the impact strength of ES-GO/BMI composites rises rapidly with the increase of ES-GO content when the ES-GO loading was small.

When the amount of ES-GO is 0.3 wt.%, the impact strength of the composite reaches a maximum of 20.57 KJ·m^−2^, which increases by 144% compared with the unmodified system. When the ES-GO content further increases, the impact strength decreases instead. The flexural strength and impact strength showed roughly the same increasing trend, because the curing reaction was centered on the curing agent, and the reaction developed from the center to the surroundings in a radial pattern during curing.

Moreover, when the content of ES-GO is less than 0.3 wt.%, ES-GO can be uniformly dispersed in the resin matrix. At this stage, it becomes part of the entire cross-linked macromolecular structure that ES-GO reacts with the resin system. It can be considered that ES-GO acts as a chain extender for BMI. With the introduction of ES-GO, the modified resin system formed a large number of cavity structures, and the distance between molecular chains increased relatively. At the same time, the modified system also formed a large number of interpenetrating network structures, which benefits the molecular segments less prone to relative slip and promotes the transmission and dispersion of stress under the action of external forces, hence resulting in an improvement of the mechanical properties of the modified system. To our knowledge, the toughening mechanism includes the plastic yielding of the matrix and subsequent void formation, and the interference of rigid groups during crack propagation, such as crack pinning and crack deflection [51,52]. (The schematic illustrations of crack pinning and crack deflection are shown in Figure 5b and Figure 5c, respectively). When the content of ES-GO is more than 0.3 wt.%, the groups that react with ES-GO at this stage have been consumed. Excess ES-GO makes its dispersion difficult, and the accumulation of ES-GO in the system agglomerates, which in turn affects the phase structure of the system. This can cause defects in the resin matrix, resulting in a decrease in the performance of the composite material.

**Figure 6:**
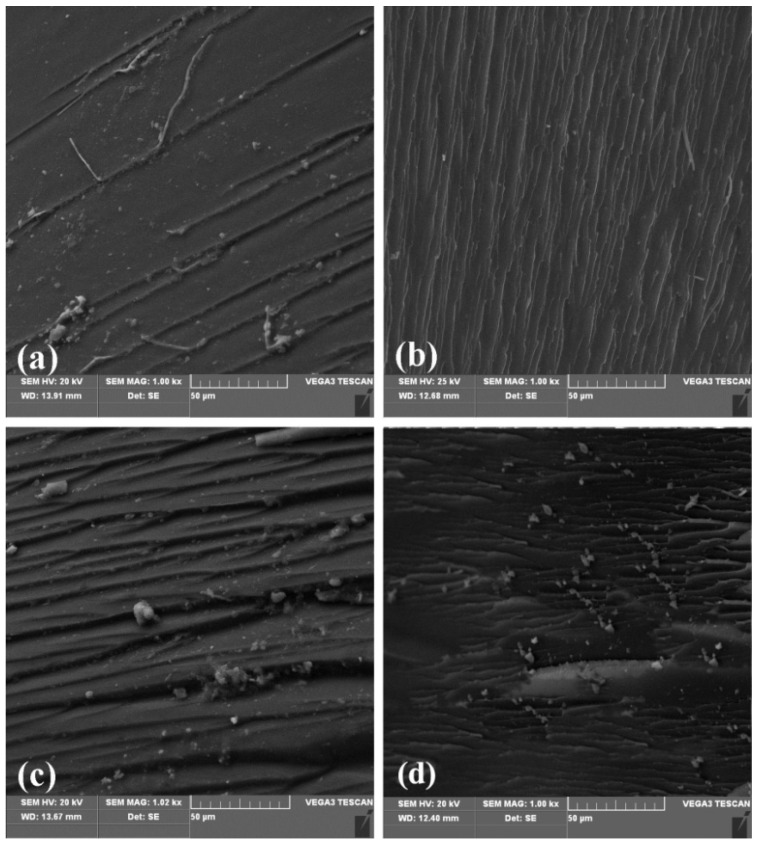
Effect of ES-GO content on the morphology of fractured surface of BMI with ES-GO contents: (**a**) 0, (**b**) 0.1 wt.%, (**c**) 0.3 wt.%, (**d**) 0.5 wt.%.

Figure 6 shows the SEM images of the fracture surface of the cured system with different ES-GO content.

It can be seen that the fracture surface of the unmodified system (Figure 6a) is relatively smooth, almost no ridges can be seen. It shows less gullies and less stress dispersion, which is a typical brittle fracture characteristic. Figure 6b is the fracture morphology of the modified system with 0.1 wt.% content of ES-GO. It can be found that the cross-section is undulating, rough and presents continuous gullies and tree branches and that there are many restrictions between cracks. The damage is not carried out in a plane, and the direction of fracture also tends to be scattered. These gullies and micro-cracks indicate that the resin casting body produced micro-plastic deformation during the fracture process. The direction of crack propagation is changed, and the energy dissipation path of the resin casting body is increased, thereby improving the impact strength. With the content of ES-GO increasing to 0.3 wt.%, as shown in Figure 6c, the fracture morphology of the corresponding system shows more and more obvious faults and ravines, and the fracture surface is rough and irregular, showing disorder; Furthermore, the fracture direction tends to be scattered, which is a process of gradually increasing in toughness. However, with the higher content of ES-GO system (0.5 wt.%), as shown in Figure 6d, the fracture surface shows relatively less streaks and shallower grooves, which implies a reduction in toughness compared with 0.3 wt.% ES-GO/BMI system. This can be attributed to a higher proportion of unreacted ES-GO accumulation; agglomeration has an adverse effect on toughness. The observation results of the fracture surface of the resin system are consistent with the change trend of mechanical properties, which proves that ES-GO can improve the toughness of the BMI system to a certain extent.

### 3.5. Damp Heat Resistance

**Figure 7:**
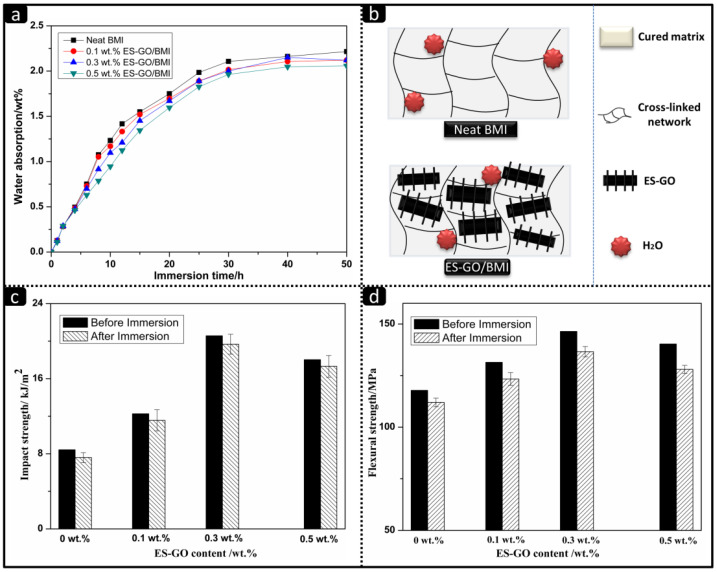
(**a**) Water absorption curves of cured resins with different content of ES-GO under boiling water; (**b**) Mechanism of water resistance; (**c**) Impact strength and (**d**) flexural strength of resin systems before and after immersing in boiling water.

The modified system presents good moisture and heat resistance due to a large number of benzene rings and other groups, in addition to a large crosslinking density and inherently few hydrophilic groups. Figure 7a shows the water absorption of different ES-GO content of cured resin systems under boiling water conditions.

It can be seen that the water absorption rates of all systems follow a typical kinetic curve time vs. concentration, with water uptake increase depending on the time. The initial stage of water absorption belongs to the diffusion control stage. Water molecules could quickly diffuse to the free space on the surface of the cured product in this stage, and the water absorption rate was determined by the concentration difference between the inside and outside of the cured product. Therefore, the water absorption rates of all systems are similar. With the increase in time of immersion, it can be seen that the ES-GO content is inversely proportional to the water absorption rate of the system. The higher the ES-GO content is, the lower the water absorption rates become, which can be attributed to the movement of the water molecule being restricted by the higher density of cross-linked network in the matrix. The schematic illustration of the mechanism of water resistance is shown in Figure 7b. Therefore, the higher the density of the cross-linked network is, the greater the restriction of the water molecule is, and the lower the water absorption rate becomes. As analyzed, when the content of ES-GO increases, the cross-link density of the modified resin system would increase and the free volume would become smaller, which results in an improvement of moisture absorption. It can be seen from Figure 7a that the saturated water absorption of the 0.5 wt.% ES-GO content system stay approximately at 2.1%.

Figure 7c,d show the impact strength and flexural strength of cured resin systems with different ES-GO contents before and after boiling for 50 h. It can be seen that the effect of wet heat aging on the mechanical properties of the cured product are not obvious. Moreover, since the moisture absorption rate of the modified system is slightly lower than that of the unmodified system, the reductions of impact strength and flexural strength of modified resin systems are slightly lower than that of the unmodified system after boiling. All results indicate that the modified resin possesses good resistance to moisture and heat.

### 3.6. Properties of Composites

In order to evaluate the application efficiency of the modified resin, glass-fiber-reinforced composites with different ES-GO loading were prepared and tested for performance as summarized in Table 2.

As can be seen, the mechanical properties of all composite systems are good for structural applications and possess the high retention rate of mechanical properties at 150 °C. With good wettability with fibers and moderate viscosity at the curing temperature, the composite systems of ES-GO/BMI possess relatively superior mechanical properties compared with a neat composite system. The mechanical properties of a composite increase by 25–35% by the addition of 0.3 wt.% ES-GO.

The effect of temperature on the properties of the composite material is mainly achieved by the effect on the resin matrix. When the temperature rises, the amorphous part of the resin matrix softens, and after that its deformation increases, which weakens the role of the matrix in the composite material, resulting in a decrease in the strength and modulus. It can be seen from Table 2 that the flexural strength and interlaminar shear strength of the composites decrease by 15–20% at 150 °C. The retention rates of properties of the unmodified system is slightly lower than that of the modified system at 150 °C. As analyzed in this article, the heat resistance of the resin matrix was improved to a certain extent by the introduction of ES-GO, hence the retention rate of properties of its composite also can be improved at high temperature. The higher the ES-GO content is, the higher the retention rate of the corresponding high-temperature performance of the composite material become.

In generally, the resin matrix possesses a certain degree of moisture absorption, therefore, the glass-fiber-reinforced composite is particularly sensitive to heat and humidity. Various characteristics of the composite would change under the action of wet stress for a certain period of time, hence the mechanical properties and dimensional stability of the composite would be affected eventually. Therefore, the moisture resistance of a composite is one of the most important parameters. It can be seen from Table 2 that the performances of all composites have decreased after boiling for 50 h. The flexural strength of the unmodified system decreases by 4% and the interlayer shear strength decrease by 6%. The modified systems possess higher retention rates in flexural strength and interlayer strength compared with an unmodified system. To our knowledge, the matrix of composites could incur internal stress due to its interface swell after boiling, which generates cracks and results in a decrease in the interface bonding force. In addition, the chemical reaction between water and glass fiber could occur after boiling, which would result in an irreversible loss in strength. In terms of fiber, the greater the content of alkali is, the faster the rate of erosion by water becomes.

Therefore, the retention rate of the boiled properties of the composite is mainly attributed to the water absorption rate of the resin matrix and the interfacial bonding force between the matrix and fiber. The above analyses show that the modified resin matrixes possess good moisture and heat resistance compared with neat system, which is consistent with the retention rate of performance of the composite after boiling. All in all, the test results of composites show that the purpose of modification is achieved.

## 4. Conclusions

A graphene oxide modified by epoxy silane has been synthesized and used to prepare a thermosetting resin with BMI resin. Various formulations containing 0 to 0.5 wt.% ES-GO were made and characterized for thermal and mechanical properties of modified resin as well as glass-fiber-reinforced composites. Characterizations including XPS and FT-IR results demonstrated that the epoxy silane molecules were successfully linked onto the GO surfaces. Due to the chemical interaction between the BMI and the functional groups of ES-GO surfaces, the thermal and mechanical properties of the ES-GO/BMI nanocomposites were improved significantly. TGA results showed that modified BMI resin systems displayed the higher char yield at various formulations containing ES-GO as compared to that neat resin. Using DMA, *Tg* was found to increase with increasing ES-GO loadings, which can be attributed to the increase of the crosslink density of the cured network. As expected, a suitable amount of ES-GO (0.3 wt.%) led to a higher packing density of mesogenic networks, hence maximized the impact and flexural strengths of the modified, which increased 144% and 20%, respectively. Moreover, when the modified BMI resin was used to laminate a glass-fiber-reinforced composite, the ES-GO/BMI systems showed better flexural and shear strengths and higher retention rates under 150 °C as compared to that made with a neat BMI.

## Figures and Tables

**Table 1:** Thermal properties of systems modified with various contents of ES-GO.

ES-GO Content (wt.%)	Thermogravimetric Analysis (TGA) Data			Tg (°C)		HDT (°C)
	T_0.05_ (°C)	T_max_ (°C)	Char Yield at 850 °C (%)	DSC	DMA	
0	446	535	28.6	287	305	269
0.1	449	566	31.2	300	313	274
0.3	448	582	32.4	310	325	290
0.5	450	590	35.6	316	332	292

**Table 2:** Properties of the composites.

ES-GO Content (wt.%)	Flexural Strength (MPa)/Retention Rate			Shear Strength (MPa)/Retention Rate		
	A	B	C	A	B	C
0	638	551/86.3%	611/95.7%	58.4	49.2/84.2%	54.7/93.6%
0.1	692	591/87.4%	656/95.8%	61.5	50.6/86.3%	56.1/94.3%
0.3	791	645/87.6%	740/96.7%	68.8	53.2/87.8%	60.8/95.4%
0.5	757	688/88.3%	793/96.5%	62.6	56.9/88.2%	65.8/95.9%

A: Room temperature; B: 150 °C; C: Immersing in boiling water for 50 h.
